# A meta-analysis of effects of vitamin E supplementation alone and in combination with omega-3 or magnesium on polycystic ovary syndrome

**DOI:** 10.1038/s41598-022-24467-0

**Published:** 2022-11-19

**Authors:** Hajar Heidari, Zahra Hajhashemy, Parvane Saneei

**Affiliations:** 1 Department of Community Nutrition, School of Nutrition and Food Science, Nutrition and Food Security Research Center, Isfahan University of Medical Sciences, PO Box 81745-151, Isfahan, Iran; 2 Students’ Scientific Research Center, Isfahan University of Medical Sciences, Isfahan, Iran

**Keywords:** Obesity, Polycystic ovary syndrome, Nutrition

## Abstract

Vitamin E supplementation might have favorable effects on risk factors of polycystic ovary syndrome (PCOS). This systematic review and meta-analysis aimed to summarize the effects of vitamin E supplementation or vitamin E in combination with omega-3 or magnesium on PCOS. PubMed, Scopus, ISI Web of Science, Cochrane, Embase electronic databases, and Google scholar were searched for all available articles up to September 2022. Randomized controlled trials (RCTs) that examined the effect of vitamin E supplementation or vitamin E in combination with omega-3 or magnesium on lipid and glycemic profiles, anthropometric measurements, biomarkers of inflammation and oxidative stress, hormonal profile, and hirsutism score in patients with PCOS were included. Ten RCTs (with 504 participants) fulfilled the eligible criteria. Vitamin E supplementation or vitamin E in combination with omega-3 or magnesium in comparison to placebo could significantly reduce serum levels of TG (weighted mean difference: − 18.27 mg/dL, 95% CI − 34.68 to − 1.87), VLDL (− 5.88 mg/dL, 95% CI − 8.08 to − 3.68), LDL-c (− 12.84 mg/dL, 95% CI − 22.15 to − 3.52), TC (− 16.30 mg/dL, 95% CI − 29.74 to − 2.86), TC/HDL-c ratio (− 0.52, 95% CI − 0.87 to − 0.18), hs-CRP (− 0.60 ng/mL, 95% CI − 0.77 to − 0.44), hirsutism score (− 0.33, 95% CI − 0.65 to − 0.02) and significantly increase nitric oxide levels (2.79 µmol/L, 95% CI 0.79–4.79). No significant effect was found on HDL-c, glycemic indices, hormonal profile, anthropometric measurements, and other biomarkers of inflammation or oxidative stress. This meta-analysis highlights the potential anti-hyperlipidemic, anti-oxidant, and anti-inflammatory properties of vitamin E supplementation alone or in combination with omega-3 or magnesium on PCOS patients.

## Introduction

Polycystic ovary syndrome (PCOS) is one of the most common and complex endocrine disturbances estimated to affect 6 to 25% of women in reproductive age^1,2^. PCOS is characterized by menstrual dysfunction, hyperandrogenism, ovulatory dysfunction, and subfertility^3^. Low-grade inflammation, including increased C-reactive protein (CRP), tumor necrosis factor-α (TNF-α), and interleukin-6 (IL-6) plays a role in pathophysiology of PCOS^4^. Hyperinsulinemia and insulin resistance seem to be predominant features of PCOS, along with dysfunction of the hypothalamic-pituitary axis, which result in hormonal alterations, including increased serum luteinizing hormone (LH)/ follicle-stimulating hormone (FSH) ratio and circulating androgens. Dyslipidemia is also present in 70% of PCOS patients^5,6^. Thus, this syndrome can result in obesity and metabolic disorders like dyslipidemia, insulin resistance and increased hormonal and inflammatory disorders, and oxidative stress. Long-term consequences of PCOS are type 2 diabetes mellitus (T2DM), cardiovascular diseases (CVD), hypertension, cancer, and psychological problems^7–9^. Therefore, the adverse effects of PCOS could threaten the quality of life in women^10^.

The etiology of PCOS is unknown, but genetics along with environmental and lifestyle factors have been significantly implied in the development of the syndrome^11,12^. Lifestyle changes and nutritional interventions along with weight loss are successful treatments for patients with PCOS^1^. Dietary factors like anti-inflammatory foods may have a significant role in improving metabolic disorders of the syndrome^10^. Recently, there was an increasing attention to the health benefits of vitamin E. Vitamin E could possess anti-inflammatory, anti-oxidative, anti-hyperglycemic, anti-hypertensive, anti-hypercholesterolemic, and anti-obesity properties^13^.

Several clinical trials have examined the effect of vitamin E supplementation alone or in combination with omega-3 or magnesium on improving metabolic and hormonal profile, inflammatory markers, oxidative stress, hirsutism, and anthropometric parameters in PCOS patients, but controversial results have been reported^2,14–23^. Some trials showed that vitamin E supplementation alone or along with omega-3 could significantly improve total and free testosterone in PCOS patients^15,16^. In contrast, others reported that vitamin E and magnesium co-supplementation did not affect serum total testosterone levels^14^. Several studies indicated that vitamin E and magnesium or omega-3 co-supplementation might improve most indicators of lipid profiles in PCOS patients^15,17^. However, Izadi et al. suggested that vitamin E supplementation could only lead to a significant decrease in serum TG levels^18^. In addition, Chen et al. showed that short-term supplementation with vitamin E could improve oxidative stress markers such as malondialdehyde (MDA)^19^; while, other trials suggested that vitamin E supplementation along with magnesium did not affect MDA levels in PCOS patients^14^. To our knowledge, no previous review has systematically summarized the effects of vitamin E supplementation alone or along with omega-3 or magnesium on improvement of the metabolic and hormonal profile, inflammatory markers, oxidative stress, hirsutism, and anthropometric parameters in PCOS. So, we conducted a systematic review and meta-analysis on randomized clinical trials that evaluated the effects of vitamin E supplementation or vitamin E along with omega-3 or magnesium supplementation on PCOS patients.

## Materials and methods

### Search strategy

A comprehensive literature search was conducted of the MEDLINE (PubMed), Scopus, ISI Web of Science, Cochrane, and Embase electronic databases, as well as Google scholar, up to September 2022, with no limitation in language, time of publication or study location. The following combination of search terms was used: (“vitamin E” OR tocopherol OR tocotrienol OR “VIT E” OR “alpha-Tocopherol” OR “beta-Tocopherol” OR “gamma-Tocopherol” OR magnesium OR omega-3 OR “n-3 fatty acid” OR W-3 OR EPA OR DHA OR ALA OR “fish oil” OR “alpha-Linolenic Acid” OR “Docosahexaenoic Acids” OR “Eicosapentaenoic Acid”) AND (PCOS OR “polycystic ovarian syndrome” OR “Polycystic Ovary Syndrome”). Moreover, manual searches of the bibliographies of the relevant investigations were performed to avoid missing any publication. Duplicate citations were removed. The article selection was independently carried out by 2 investigators (H.H. and Z.H.), and any disagreement was resolved by consultation with the principal investigator (P.S.). The Preferred Reporting Items for Systematic Reviews and Meta-Analyses guideline (PRISMA) were followed in the present report. The study protocol was registered at PROSPERO (no. CRD42021256820).

### Inclusion and exclusion criteria

Published articles were included in the systematic review and meta-analysis if they met the following criteria: (1) be a randomized controlled trial (RCT); (2) conducted on women with PCOS; (3) supplemented intervention group with vitamin E or vitamin E in combination with omega-3 or magnesium, and control group with placebo; and (4) reported means and standard deviations (SDs) or standard errors (SEs) of lipid profile (TG, very low-density lipoprotein-cholesterol (VLDL), LDL-c, HDL-c, TC and TC/HDL-c ratio), glycemic indices (fasting blood sugar (FBS), insulin, homeostasis model assessment-estimated insulin resistance (HOMA-IR) and quantitative insulin sensitivity check index (QUICKI)), hormonal parameters (total testosterone, serum luteinizing hormone (LH), follicle stimulating hormone (FSH), sex hormone-binding globulin (SHB) and free androgen index (FAI)), biomarkers of inflammation and oxidative stress (total antioxidant capacity (TAC), glutathione (GSH), MDA, high sensitive C-reactive protein (hs-CRP) and nitric oxide (NO)), anthropometric measurements (weight, body mass index (BMI), waist circumference and metabolic equivalents (METs)), and/or hirsutism score (ferriman–Gallwey (mf-G)), before and after supplementation. Studies were excluded if they did not provide integrated data or did not have an appropriate intervention. Details of more related studies that were excluded from the present review are described in Supplemental Table S1.

### Data extraction

The following data were extracted from the eligible papers: first author’s name, publication year, study location, sample size of each intervention group, age range or mean age, number of participants in each group, study design, duration of intervention, dosage of vitamin E supplementation or vitamin E in combination with omega-3 or magnesium, type of intervention, and mean ± SD/SE of lipid and glycemic profiles, anthropometric measurements, biomarkers of inflammation and oxidative stress, hormonal profile and hirsutism score at baseline and the end of the intervention, matching of two intervention groups and final adjustments in the analysis. This process was independently performed by two investigators (H.H. and Z.H.). The principal researcher (P.S.) supervised data extraction. All reported SEs were converted to SDs using the appropriate formula. When the concentration of an indicator was reported in different units among included studies, we converted them to the most frequently used unit.

### Quality assessment of studies

Revised Cochrane Collaboration's tools (RoB2.0) was used to assess the quality of each included RCT^24^. According to this tool, each trial was assessed based on the following domains: random sequence generation (selection bias), allocation concealment (selection bias), blinding of participants and personnel (performance bias), blinding of outcome assessment (detection bias), incomplete outcome data (attrition bias), selective reporting (reporting bias), and other sources of bias. For each study, two authors have independently evaluated the risk of bias as low risk, high risk, and some concern for each domain. Finally, the overall quality of the study was categorized into: low risk, if the study was judged to be at low risk of bias for all domains; high risk, if the study was judged to be at high risk of bias in at least one domain; some concern, if the study was judged to be at some concern in at least one domain^24^.

### Statistical analysis

To pool the effect of vitamin E supplementation or vitamin E in combination with omega-3 or magnesium supplementation on women with PCOS, the mean change and its standard deviation for intervention and control groups were extracted or calculated. Then, weighted mean differences (WMDs) with 95% confidence intervals (CIs) were computed through a random effects model. Between-study heterogeneity was tested by Cochran's Q test and quantified by I^2^ statistic. Subgroup analysis and meta-regression were performed to find the source of heterogeneity. Sensitivity analysis was used to explore the influence of a single study on the overall estimate. Publication bias was examined via visual inspection of funnel plots. A formal statistical assessment of funnel plot asymmetry was done using Begg’s and Egger’s tests. All statistical analyses were performed using STATA, version 11.2 (STATA Corp., College Station, TX). P values less than 0.05 were considered as statistically significant.

## Results

### Selection and identification of studies

**Figure 1:**
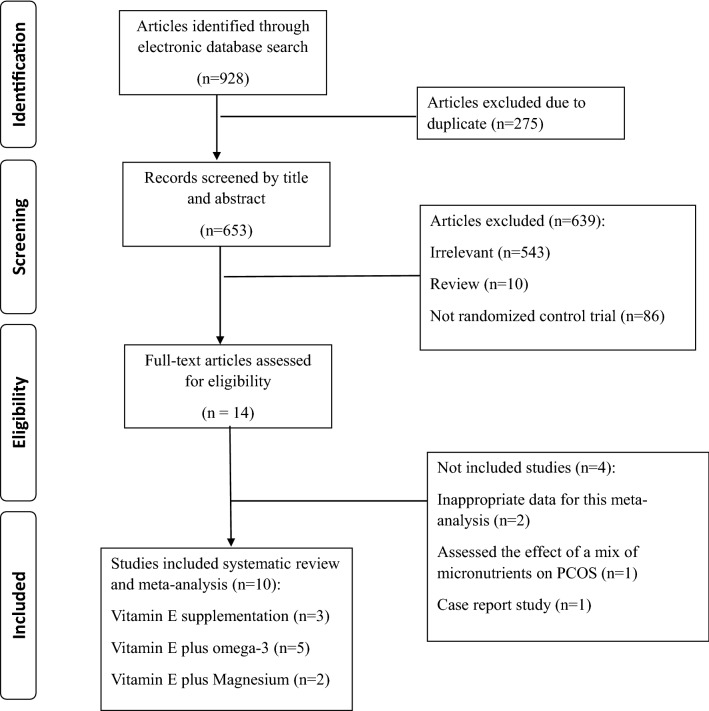
Flow chart of the systematic search and study selection.

Out of the initial 928 studies obtained by electronic and manual searches, 275 articles were excluded as duplicates. First, the title and abstract of the remaining 653 articles were screened, and 639 of them were excluded based on the inclusion criteria. Then, the full text of 14 articles was carefully assessed, and after excluding 4 irrelevant studies, 10 eligible RCTs were included in the present meta-analysis. It should be noted that Izadi et al. have conducted a trial on 43 women with PCOS and reported the results in two papers^16,18^; one of these papers showed the effect of vitamin E supplementation on lipid profile, and the other one reported the impact of vitamin E supplementation on glycemic and hormonal profiles. So, there was no overlapping in outcomes of interest, and we considered these reports as two trials. Finally, 10 RCTs were included in the present meta-analysis. A flow chart describing the systematic search and study selection process is illustrated in Fig. 1.

### Systematic review

The main characteristics of 10 RCTs included in the systematic review and meta-analysis are described in Table 1. All eligible RCTs were carried out in Iran, published between 2017 and 2019, and had a sample size ranging from 40 to 68 participants. The mean age of participants in these trials ranged from 18 to 40 years. Duration of the intervention period varied from 8 to 12 weeks. In all RCTs, PCOS was defined based on the 2003 Rotterdam criteria. All RCTs had a parallel study design. The intervention group received vitamin E supplement in 3 trials^2,16,18^, vitamin E plus magnesium in 2 studies^14,17^, and vitamin E plus omega-3 in 5 studies^15,20–23^, while in all studies the control group received placebo. The dose of vitamin E for the intervention group ranged from 400 to 888 IU/day. Doses of omega-3 and magnesium co-supplemented with vitamin E in RCTs were 1000 mg/day and 250 mg/day, respectively. These trials reported the effects of vitamin E supplementation or vitamin E in combination with omega-3 or magnesium supplementation on anthropometric measurements, lipid and glycemic profiles, biomarkers of inflammation and oxidative stress, hormonal profile and hirsutism score.

**Table 1:** The main characteristics of clinical trial studies which were included in the systematic review and meta-analysis.

Author, year	Country	Mean age/age range (year) (intervention/control)	No. of participants (intervention/control)	Study duration (wk)	Control	Intervention (content/dose)	Outcome		Intervention			Control			Adjustment or matching	Quality score^1^
									Before (mean ± SD)	After (mean ± SD)	Change (SD/SE/95% CI)	Before (mean ± SD)	After (mean ± SD)	Change (SD/SE/95% CI)		
Jamilian, 2018	Iran	18–40 (22.3 ± 4.7/24.4 ± 4.7 )	40 (20/20)	12	Placebo (paraffin)	1000 mg omega-3 fatty acids plus 400 IU vitamin E supplements	Anthropometric measurements	Weight(kg)	73.6 ± 11.7	72.7 ± 11.8	− 0.9 ± 1.5	69.8 ± 17.1	69.4 ± 16.9	− 0.4 ± 1.1	Matched for age and BMI	6
								BMI(kg/m^2^)	28.8 ± 5.1	28.5 ± 5.1	− 0.3 ± 0.6	26.5 ± 5.9	26.3 ± 5.8	− 0.2 ± 0.4		
								HC (cm)	102.1 ± 12.1	101.7 ± 13.1	− 0.4 ± 0.5	98.7 ± 12.6	98.6 ± 12.4	− 0.1 ± 1.0		
								WC (cm)	90.0 ± 12.7	89.6 ± 12.6	− 0.4 ± 0.5	87.1 ± 12.4	86.9 ± 12.2	− 0.2 ± 0.6		
								METs	27.9 ± 2.1	28.0 ± 2.2	0.1 ± 0.9	27.1 ± 1.9	27.2 ± 2.1	0.1 ± 0.6		
							Hirsutism	mF-G	15.8 ± 4.0	15.1 ± 3.6	− 0.7 ± 0.9	14.1 ± 3.6	13.9 ± 3.3	− 0.2 ± 0.5		
Rahmani, 2017	Iran	18–40 (24.9 ± 5.5/26.6 ± 5.6 )	68 (34/34)	12	Placebo	1000 mg omega-3 fatty acids from flaxseed oil containing 400 mg α-linolenic acid plus 400 IU vitamin E supplements	Anthropometric measurements	Weight(kg)	74.1 ± 10.7	73.8 ± 10.8	− 0.3 ± 1.1	77.6 ± 18.2	77.4 ± 18.3	− 0.2 ± 1.1	Matched for age and phenotypes A and D of PCOS	7
								BMI(kg/m^2^)	28.4 ± 4.4	28.2 ± 4.6	− 0.1 ± 0.4	29.0 ± 6.5	29.0 ± 6.5	− 0.1 ± 0.4		
								METs	29.4 ± 2.5	29.5 ± 2.5	0.1 ± 0.8	28.9 ± 2.3	28.8 ± 2.3	− 0.1 ± 0.8		
							Hormonal profile	FSH (IU/L)	7.3 ± 2.5	7.2 ± 2.5	− 0.4 ± 0.5*	7.9 ± 2.8	8.1 ± 3.2	0.5 ± 0.5*	Adjusted for baseline values, age and BMI at baseline	
								LH (IU/L)	11.0 ± 8.0	10.5 ± 8.9	− 1.2 ± 1.4*	13.5 ± 13.3	11.4 ± 7.7	− 1.5 ± 0.5*		
							oxidative stress	TAC (mmol/L)	860.5 ± 101.0	949.9 ± 119.3	60.3 ± 19.1*	969.5 ± 85.3	975.4 ± 98.0	35 ± 19.2*		
								GSH (µmol/L)	525.3 ± 84.1	544.8 ± 81.3	21.7 ± 8.7*	511.8 ± 69.1	555.2 ± 62.4	41.1 ± 8.7*		
								MDA (µmol/L)	2.9 ± 0.6	2.5 ± 0.6	− 0.2 ± 0.1*	2.2 ± 0.5	2.2 ± 0.5	− 0.2 ± 0.1*		
							Biochemical profile	TG (mg/dL)	122.7 ± 61.7	100.6 ± 54.0	− 21.9 ± 4*	120.6 ± 59.4	128.3 ± 72.6	7.5 ± 4*		
								VLDL-C (mg/dL)	24.5 ± 12.3	20.1 ± 10.8	− 4.4 ± 0.8*	24.1 ± 11.9	25.7 ± 14.5	1.5 ± 0.8*		
								TC(mg/dL)	181.8 ± 28.0	161.5 ± 31.4	− 18.1 ± 3.8*	166.4 ± 29.2	178.6 ± 29.9	10 ± 3.8*		
								LDL-c (mg/dL)	111.1 ± 26.5	94.4 ± 29.8	− 13.3 ± 3.7*	92.9 ± 25.5	104.8 ± 26.3	8.5 ± 3.7*		
								HDL-C (mg/dL)	46.2 ± 10.0	47.0 ± 9.5	0.2 ± 0.8*	49.4 ± 8.1	48.1 ± 9.3	− 0.7 ± 0.8*		
								TC/HDL-c	4.1 ± 1.0	3.6 ± 0.9	− 0.4 ± 0.1*	3.5 ± 0.8	3.9 ± 1.1	0.3 ± 0.1*		
Ebrahimi, 2017	Iran	18–40 (23.8 ± 4.6/25.2 ± 5.2 )	68 (34/34)	12	Placebo	1000 mg omega-3 fatty acids from flaxseed oil containing 400 mg α-Linolenic acid plus 400 IU vitamin E supplements	Anthropometric measurements	Weight (kg)	72.4 ± 10.7	71.9 ± 10.7 0	− 0.5 ± 1.3	75.1 ± 18.2	74.8 ± 18.3	− 0.3 ± 1.1	Matched for age, BMI and phenotypes A and D of PCOS	7
								BMI(kg/m^2^)	28.0 ± 4.3	27.8 ± 4.3	− 0.2 ± 0.5	28.5 ± 6.6	28.3 ± 6.7	− 0.2 ± 0.4		
								METs	27.0 ± 2.3	27.0 ± 2.4	0.02 ± 0.7	26.8 ± 2.1	26.7 ± 2.2	− 0.1 ± 0.7		
							Biochemical profile	FPG(mg/dL)	90.2 ± 10.2	87.0 ± 8.6	− 3.8 ± 1.1*	94.8 ± 7.4	94.1 ± 9.1	− 0.05 ± 1.1*	Adjusted for baseline values, age and BMI at baseline	
								Insulin (μIU/mL)	10.8 ± 4.8	9.8 ± 4.9	− 0.8 ± 0.9*	9.8 ± 5.7	12.5 ± 6.6	2.5 ± 0.9*		
								HOMA-IR	2.4 ± 1.2	2.2 ± 1.2	− 0.2 ± 0.2*	2.3 ± 1.4	2.9 ± 1.6	0.6 ± 0.2*		
								HOMA-B	39.7 ± 18.6	35.4 ± 19.1	− 3.0 ± 3.3*	33.7 ± 21.4	44.1 ± 25.4	9.2 ± 3.3*		
								QUICKI	0.34 ± 0.02	0.34 ± 0.02	0.002 ± 0.005*	0.35 ± 0.04	0.33 ± 0.02	− 0.01 ± 0.005*		
							Hirsutism	mF-G	13.6 ± 3.7	13.3 ± 3.7	− 0.4 ± 0.2*	12.2 ± 3.5	12.0 ± 3.4	− 0.3 ± 0.2*		
							Hormonal profile	Total testosterone (ng/mL)	1.2 ± 0.9	0.7 ± 0.6	− 0.4 ± 0.08*	1.1 ± 0.6	1.0 ± 0.6	− 0.06 ± 0.08*		7
								Free testosterone (pg/mL)	4.5 ± 3.2	3.3 ± 2.4	− 1.1 ± 0.3*	3.9 ± 2.7	3.7 ± 2.3	− 0.4 ± 0.3*		
								SHBG (nmol/L)	37.5 ± 15.9	44.1 ± 21.3	6.9 ± 2.4*	39.1 ± 15.0	44.9 ± 16.9	5.5 ± 2.4*		
								FAI	0.14 ± 0.13	0.09 ± 0.11	− 0.04 ± 0.01*	0.12 ± 0.17	0.08 ± 0.05	− 0.04 ± 0.01*		
								DHEAS	4.5 ± 2.3	3.5 ± 2.0	− 1.2 ± 0.2*	5.2 ± 1.9	4.3 ± 1.5	− 0.7 ± 0.2*		
Izadi, 2019	Iran	20–40 (27.18 ± 5.77/ 26.0 ± 4.53)	43 (22/21)	8	Placebo	400 IU vitamin E	Anthropometric measurements	BMI(kg/m^2^)	29.28 ± 4.24	28.92 ± 4.23	‒0.37 (‒0.60, ‒0.14)**	28.73 ± 3.39	28.74 ± 2.9	0.01 (‒0.23, 0.25)**	–	7
								WC (cm)	95.00 ± 10.82	92.18 ± 10.94	‒2.81, (‒3.47, ‒2.15)**	89.33 ± 7.97	88.43 ± 8.04	‒0.89, (‒1.57, ‒0.21)**		
							Biochemical profile	TG (mg/dL)	111.68 ± 44.41	105.18 ± 8.22	− 6.48 (− 9.31, − 3.66)**	112.86 ± 42.27	112.09 ± 9.09	− 0.99 (− 3.89, 1.90)**	Adjusted for baseline levels, age, physical activity, dietary intake of energy and vitamin E	
								TC (mg/dL)	163.41 ± 21.86	159.00 ± 18.96	− 4.07 (− 9.14, 1.00)**	157.43 ± 18.46	159.67 ± 22.87	2.37 (− 2.84, 7.57)**		
								LDL-C (mg/dL)	82.53 ± 20.51	78.10 ± 19.83	− 4.02 (− 9.60, 1.54)**	79.57 ± 24.17	82.53 ± 22.84	3.07 (− 2.64, 8.78)**		
								HDL-C (mg/dL)	58.54 ± 9.21	59.86 ± 8.45	1.24 (− 0.56, 3.05)**	55.28 ± 11.94	54.71 ± 9.81	− 0.5 (− 2.36, 1.35)**		
								Non-HDL-C (mg/dL)	104.86 ± 22.29	99.14 ± 19.78	− 5.32 (− 10.94, 0.31)**	102.14 ± 24.39	104.95 ± 24.79	2.87 (− 2.90, 8.64)**		
Jamilian, 2019	Iran	18–40 (29.2 ± 7.2/28.3 ± 3.8 )	60 (30/30)	12w	Placebo	250 mg/day magnesium plus 400 mg/ day vitamin E supplements	Anthropometrics measurements	Weight(kg)	66.7 ± 9.5	66.6 ± 9.5	–0.1 ± 0.3	67.8 ± 10.9	67.7 ± 11.1	–0.1 ± 0.8	–	7
								BMI(kg/m^2^)	25.5 ± 3.5	25.5 ± 3.3	–0.03 ± 0.1	26.0 ± 4.7	26.0 ± 4.7	–0.05 ± 0.3		
							Biochemical profile	FPG(mg/dl)	92.1 ± 12.2	90.9 ± 11.9	–1.5 ± 1.0*	93.7 ± 5.8	94.4 ± 6.5	1.0 ± 1.0*	Adjusted for baseline values + age and baseline BMI	
								TG (mg/dl)	125.0 ± 53.0	110.0 ± 55.0	− 15.1 ± 4.4*	128.1 ± 60.6	134.7 ± 68.9	6.8 ± 4.4*		
								VLDL-C (mg/dl)	25.0 ± 10.6	22.0 ± 11.0	–3.0 ± 9.9*	25.6 ± 12.1	26.9 ± 13.8	1.3 ± 0.9*		
								TC(mg/dl)	181.6 ± 40.4	174.5 ± 32.2	–7.2 ± 4.4*	185.0 ± 34.4	193.2 ± 33.7	8.3 ± 4.4*		
								LDL-C (mg/dl)	104.5 ± 36.0	101.4 ± 30.4	–2.7 ± 4.6*	106.2 ± 37.1	114.2 ± 38.9	7.6 ± 4.6*		
								HDL-C (mg/dl)	52.1 ± 10.1	51.1 ± 8.6	–1.1 ± 1.2*	53.1 ± 9.3	52.0 ± 10.9	–1.0 ± 1.2*		
								TC/HDL-C ratio	–	–	–0.05 ± 0.1*	–	–	0.3 ± 0.1*		
								Insulin (μIU/mL)	13.4 ± 5.8	12.3 ± 5.0	–1.0 ± 0.5*	12.2 ± 5.1	13.9 ± 4.5	1.5 ± 0.5*		
								HOMA-IR	3.0 ± 1.4	2.8 ± 1.2	− 0.2 ± 0.1*	2.8 ± 1.2	3.2 ± 1.1	0.4 ± 0.1*		
								QUICKI	0.32 ± 0.01	0.33 ± 0.01	0.003 ± 0.003*	0.33 ± 0.02	0.32 ± 0.01	− 0.008 ± 0.003*		
Izadi, 2019	Iran	20–40 (27.18 ± 5.77/ 26.0 ± 4.53)	43 (22/21)	8	Placebo	400 IU vitamin E	Anthropometric measurements	Weight (kg)	76.95 ± 10.61	75.96 ± 10.3	− 1.33 (− 0.4 to 2.29)**	73.23 ± 7.58	73.29 ± 7.3	0.15 (− 0.49 to 0.9)**	–	7
							Biochemical profile	FBS(mg/dl)	85.50 ± 20.28	81.18 ± 10.28	4.32 (0.85 to 7.67)**	17.95 ± 9.25	80.57 ± 8.96	− 0.10 (− 3.69 to 3.48)**	Adjusted for baseline values, age, BMI, and physical activity	
								Insulin (mIU/L)	13.72 ± 5.92	11.44 ± 4.57	2.26 (0.36 to 4.16)**	13.47 ± 9.73	12.47 ± 7.73	0.89 (1.14 to 2.78)**		
								HOMA-IR	2.80 ± 1.17	2.35 ± 1.01	0.45 (0.02 to 0.87)**	2.73 ± 2.12	2.55 ± 1.70	0.16 (− 0.28 to 0.60)**		
							Hormonal profile	Total testosterone (ng/ml)	1.16 ± 0.40	0.84 ± 0.23	0.32 (0.17 to 0.46)**	1.33 ± 0.35	1.47 ± 0.39	− 0.13 (− 0.28 to 0.02)**		
								SHBG	38.00 (25.32 to 72.50)***	54.10 (31.50 to 66.20)***	− 5.93 (− 16.08 to 4.23)**	42.30 (25.20 to 56.80)***	40.80 (31.00 to 44.50)***	1.05 (− 9.43 to 11.52)**		
								FAI	3.08 (1.57 to 8.13)***	1.66 (1.14 to 2.92)***	1.39 (0.47 to 2.32)**	2.80 (2.02 to 4.99)***	3.53 (2.57 to 5.12)***	0.42 (− 0.91 to 0.99)**		
								LH(mIU/ml)	11.15 (7.30 to 17.05)***	7.25 (6.35 to 15.00)***	4.88 (2.72 to 7.4)**	8.40 (5.200 to 17.85)***	10.80 (6.70 to 17.95)***	− 0.49 (− 2.72 to 1.73)**		
								FSH (mIU/mL)	5.90 (5.27 to 6.77)***	5.10 (3.75 to 6.27)***	0.63 (− 0.46 to 1.72)**	7.30 (3.70 to 7.65)***	5.90 (4.80 to 7.10)***	0.21 (− 0.98 to 1.4)**		
								Progesterone (ng/mL)	1.80 ± 0.86	2.46 ± 1.02	− 0.0 (− 1.06 to − 0.27)**	1.62 ± 0.99	1.60 ± 1.12	− 0.02 (− 0.45 to 0.41)**		
								Estradiol (pg/mL)	85.45 ± 17.79	99.66 ± 23.01	− 13.92 (− 34.25 to 6.42)**	74.43 ± 17.95	71.09 ± 12.38	1.74 (− 20.36 to 23.85)**		
Talari, 2018	Iran	18–40	60 (30/30)	12	Placebo	1000 mg omega-3 plus 400 IU vitamin E supplements	Inflammatory markers	hs-CRP (ng/mL)	2877.9 ± 2095.5	2487.3 ± 1673.1	− 360.2 ± 140.1	2646.7 ± 1492.3	2883.7 ± 1488.9	206.6 ± 140.1	Adjusted for baseline values, age, BMI at baseline	*6*
								NO(μmol/L)	49.6 ± 2.3	51.3 ± 4.7	1.8 ± 0.7	46.0 ± 6.0	46.1 ± 5.9	− 0.05 ± 0.7		
Shokrpour , 2019	Iran	18–40 (27.2 ± 7.1/26.0 ± 3.7)	60 (30/30)	12w	Placebo	250 mg/day magnesium plus 400 mg/day vitamin E supplements	Anthropometric measurements	Weight (kg)	69.4 ± 10.7	69.2 ± 10.6	− 0.2 ± 0.3	70.9 ± 10.3	70.7 ± 10.4	− 0.1 ± 0.6	Matched for age and BMI	7
								BMI(kg/m^2^)	27.1 ± 4.2	27.0 ± 4.1	− 0.1 ± 0.1	27.9 ± 4.2	27.8 ± 4.2	− 0.1 ± 0.2		
							Hirsutism	mF-G	14.9 ± 2.9	14.6 ± 2.5	–	13.8 ± 3.8	13.8 ± 3.8	–	–	
							Inflammatory markers	hs-CRP (ng/mL)	3.7 ± 1.9	3.1 ± 1.7	–	3.5 ± 1.5	3.7 ± 1.5	–		
								NO(μmol/L)	34.4 ± 2.3	38.7 ± 4.0	–	36.6 ± 5.6	37.0 ± 5.8	–		
							Oxidative stress	TAC (mmol/l)	522.4 ± 30.6	590.7 ± 52.2	–	513.7 ± 81.7	514.5 ± 77.3	–		
								GSH (μmol/L)	508.1 ± 69.1	519.4 ± 47.7	–	481.1 ± 101.2	483.8 ± 94.2	–		
								MDA (μmol/L)	2.7 ± 0.2	2.6 ± 0.2	–	2.4 ± 0.5	2.5 ± 0.5	–		
							Hormonal profile	Total testosterone (ng/ml)	1.4 ± 0.8	1.3 ± 0.7	–	1.2 ± 0.5	1.2 ± 0.6	–		
								SHBG )nmol/l)	51.4 ± 26.4	62.9 ± 36.3	–	48.5 ± 15.1	49.2 ± 15.2	–		
								FAI	0.15 ± 0.16	0.11 ± 0.11	–	0.09 ± 0.05	0.09 ± 0.05	–		
Sadeghi, 2019	Iran	18–40 (26.67 ± 3.35 /26.98 ± 3.78)	62 (32/30)	8w	Placebo	2 g of omega-3 plus 400 IU of vitamin E	Oxidative stress	TAC(mg/dl)	12.42 ± 1.95	13.58 ± 2.06	1.15 ± 0.93	12.22 ± 1.91	12.16 ± 1.96	− 0.6 ± 0.72	–	6
								Catalase (IU/L)	10.18 ± 1.27	12.01 ± 1.26	1.19 ± 1.06	11.14 ± 1.11	11.26 ± 1.15	0.12 ± 0.36		
								GLU (μmol/L)	10.65 ± 2.57	12.15 ± 2.66	1.5 ± 1.06	10.77 ± 2.53	11.00 ± 2.65	0.23 ± 1.43		
								MDA (μmol/L)	1.76 ± 0.29	1.42 ± 0.26	− 0.34 ± 0.32	1.38 ± 0.26	1.95 ± 2.23	0.57 ± 2.20		
Shirazi, 2019	Iran	20–40 (27.18 ± 5.77 /26.0 ± 4.53)	43 (22/21)	8w	Placebo	400 IU/day vitamin E -as alpha tocopheryl acetate	Anthropometric measurements	Weight (kg)	76.95 ± 10.61	75.96 ± 10.3	–	73.23 ± 7.58	73.29 ± 7.3	–	Matched for age and BMI	7
								BMI(kg/m^2^)	29.45 ± 5.35	29.07 ± 5.16	–	28.80 ± 3.71	28.83 ± 3.70	–		

*BMI* body mass index, *WC* waist circumference, *HC* hip circumference, *METs* metabolic equivalents, *FBS* fasting blood sugar, *FPG* fasting plasma glucose, *HOMA-β* the homeostasis model assessment-β cell function, *HOMA-IR* the homeostasis model, *QUICKI* quantitative insulin sensitivity check index, *TG* triglyceride, *HDL-c* high density lipoprotein cholesterol, *LDL-c* low density lipoprotein cholesterol, *VLDL-c* very low-density lipoprotein-cholesterol, *TC* TCesterol, *TAC* total antioxidant capacity, *NO* nitric oxide, *hs-CRP* high sensitive C-reactive protein, *MDA* malondialdehyde, *GSH* glutathione, *LH* serum luteinizing hormone, *FSH* follicle stimulating hormone, *FAI* free androgen index, *SHBG* sex hormone-binding globulin, *DHEAS* dehydroepiandrosterone sulfate, *GLU* glutathione, *mF-G* ferriman–Gallwey.

^1^According to Cochrane quality score.

*SE.

** 95% CI.

***IQR.

In case of sample size of the included RCTs, considering a type one error (α) of 5% and power of 80 or 90%, along with HOMA-IR, TG, hs-CRP and progesterone as the key outcome variables, sample size for each intervention group was calculated by the use of the proposed formula for parallel clinical trials. In almost all studies, an acceptable sample size was included in the final analysis^2,14–18,20,22,23^.

### Quality of included studies

Details of quality assessment of included articles are presented in Supplemental Table S2. All eligible RCTs have adequately performed random sequence generation; however, one RCT (10%) was judged as having some concern for allocation concealment^20^. All RCTs have adequately done blinding of participants and personnel and blinding of outcome assessment. One RCT (10%) was judged as high risk of bias for incomplete outcome data^21^, and one RCT (10%) was judged as high risk of bias for selective outcome reporting^22^. All RCTs showed a low risk for other sources of bias. Overall, seven RCTs (70%) were rated as low risk of bias^2,14–18,23^, and two others (20%) were rated as having high certainty of bias^21,22^, due to incomplete outcome data and selective outcome reporting and the last RCT (10%) was rated as some concern^20^.

### Meta-analysis of the effects of vitamin E supplementation or vitamin E along with omega-3 or magnesium supplementation on lipid profile

**Figure 2:**
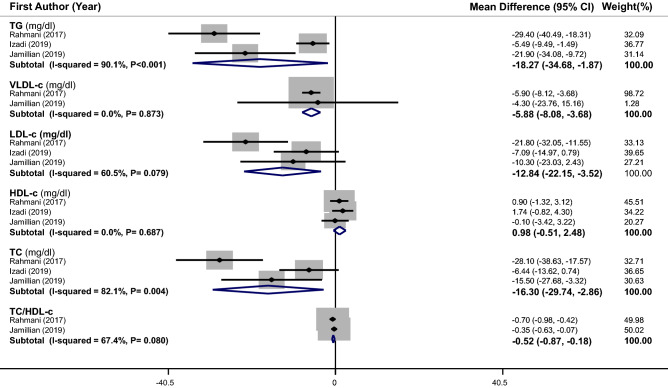
Forest plots of the effect of vitamin E supplementation or vitamin E along with omega-3 or magnesium supplementation on lipid profile. If the diamond does not touch the vertical line (or the line of null effect), the overall effect is statistically significant. Significant reductions were found in serum levels of TG (WMD: − 18.27 mg/dL, 95% CI − 34.68 to − 1.87), VLDL (WMD: − 5.88 mg/dL, 95% CI − 8.08 to − 3.68), LDL-c (WMD: − 12.84 mg/dL, 95% CI − 22.15 to − 3.52), TC (WMD: − 16.30 mg/dL, 95% CI − 29.74 to − 2.86) and TC /HDL-c ratio (WMD: − 0.52, 95% CI − 0.87 to − 0.18) in PCOS patients after vitamin E supplementation or vitamin E along with omega-3 or magnesium supplementation in comparison to placebo.

The effect of supplementation on lipid profile in PCOS patients was examined in 3 RCTs^17,18,23^ (with 171 subjects). Overall, we found a significant reduction in serum levels of TG (weighted mean difference (WMD): − 18.27 mg/dL, 95% CI − 34.68 to − 1.87), VLDL (WMD: − 5.88 mg/dL, 95% CI − 8.08 to − 3.68), LDL-c (WMD: − 12.84 mg/dL, 95% CI − 22.15 to − 3.52), TC (WMD: − 16.30 mg/dL, 95% CI − 29.74 to − 2.86) and TC /HDL-c ratio (WMD: − 0.52, 95% CI − 0.87 to − 0.18) after vitamin E supplementation or vitamin E along with omega-3 or magnesium supplementation in comparison to placebo, while the observed increase in HDL-c level was not statistically significant (Fig. 2). There was a significant heterogeneity between studies in the case of TG (I^2^ = 90.1%, P < 0.001) and TC (I^2^ = 82.1%, P = 0.004). To find the source of heterogeneity, meta-regression was conducted based on age and duration of intervention. Between-study heterogeneity was removed after these meta-regressions for age (for TG: β = − 0.008, P = 0.98, I^2^ residual = 0.00%; for TC: β = 0.028, P = 0.94, I^2^ residual = 0.00%) and duration of intervention (for TG: β = − 0.187, P = 0.55, I^2^ residual = 0.00%; for TC: β = − 0.194, P = 0.63, I^2^ residual = 0.00%), although the regression coefficients were not statistically significant. Subgroup analysis could not be performed, due to the small number of eligible trials.

### Meta-analysis of the effects of vitamin E supplementation or vitamin E along with omega-3 or magnesium supplementation on biomarkers of inflammation and oxidative stress

**Figure 3:**
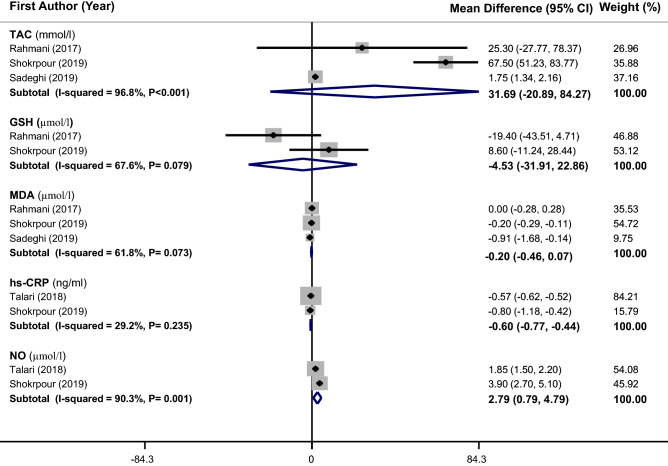
Forest plots of the effect of vitamin E supplementation or vitamin E along with omega-3 or magnesium supplementation on biomarkers of inflammation and oxidative stress. If the diamond does not touch the vertical line (or the line of null effect), the overall effect is statistically significant. A significant decrease in serum level of hs-CRP (WMD − 0.60 ng/mL, 95% CI − 0.77 to − 0.44) and a significant increase in the serum level of NO (WMD: 2.79 µmol/L, 95% CI 0.79–4.79) were found in PCOS patients after vitamin E supplementation or vitamin E along with omega-3 or magnesium supplementation in comparison to placebo.

A total of 3 RCTs^14,21,23^ with 190 participants were included in this analysis. Meta-analysis could not show any beneficial effect for vitamin E supplementation or vitamin E along with omega-3 or magnesium supplementation on TAC (WMD: 31.69 mmol/L, 95% CI − 20.89 to 84.27), GSH (WMD: − 4.53 µmol/L, 95% CI − 31.91 to 22.86) and MDA (WMD: − 0.20 µmol/L, 95% CI − 0.46 to 0.07). However, a slight but significant decrease in hs-CRP (WMD: − 0.60 ng/mL, 95% CI − 0.77 to − 0.44) and a significant increase in NO levels (WMD: 2.79 µmol/L, 95% CI 0.79–4.79) were found (Fig. 3). There was a significant heterogeneity among trials in case of TAC (I^2^ = 96.8%, P < 0.001) and NO (I^2^ = 90.3%, P = 0.001). For TAC, meta-regression based on age (β = − 0.003, P = 0.98, I^2^ residual = 0.00%) and duration of intervention (β = 0.062, P = 0.71, I^2^ residual = 0.00%) removed the observed heterogeneity, although regression coefficients for these covariates were not significant.

### Meta-analysis of the effects of vitamin E supplementation or vitamin E along with omega-3 or magnesium supplementation on glycemic indices

**Figure 4:**
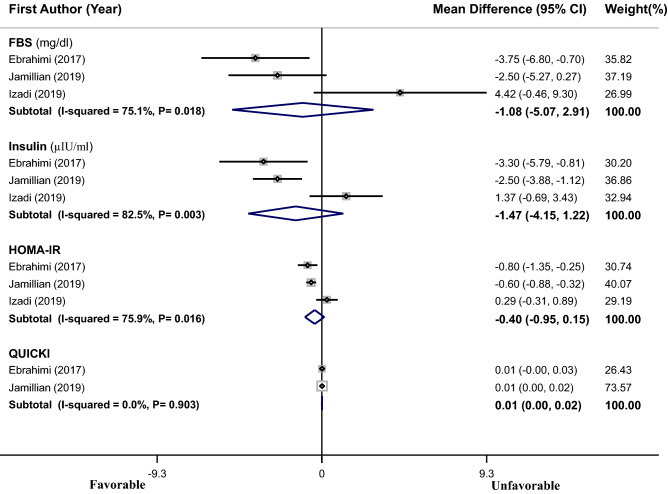
Forest plots of the effect of vitamin E supplementation or vitamin E along with omega-3 or magnesium supplementation on glycemic indexes. If the diamond does not touch the vertical line (or the line of null effect), the overall effect is statistically significant. No significant effects of vitamin E supplementation or vitamin E supplementation plus omega-3 or magnesium on glycemic indices were found in PCOS patients.

As shown in Fig. 4, pooling effect sizes from 3 RCTs^15–17^ with 171 subjects showed a non-significant reduction in FBS (WMD = − 1.08 mg/dL, 95% CI − 5.07 to 2.91), insulin (WMD = − 1.47 µIU/mL, 95% CI − 4.15 to 1.22), HOMA-IR (WMD = − 0.40, 95% CI − 0.95 to 0.15) and no change in QUICKI (WMD = 0.01, 95% CI 0.00–0.02) in PCOS patients after vitamin E supplementation or vitamin E along with omega-3 or magnesium supplementation, as compared to placebo. A significant heterogeneity was found among the studies in case of FBS (I^2^ = 75.1%, P = 0.018), insulin (I^2^ = 82.5%, P = 0.003) and HOMA-IR (I^2^ = 75.9%, P = 0.016). Meta-regression was performed to find the probable effect of age and duration of the intervention (as covariates) on heterogeneity. Findings showed that age (for FBS: β = 0.079, P = 0.91, I^2^ residual = 74.89%; for insulin: β = 0.101, P = 0.92, I^2^ residual = 89.14; for HOMA-IR: β = 0.601, P = 0.90, I^2^ residual = 99.49%) could not significantly explain the observed heterogeneity, but duration of intervention (for FBS: β = − 0.510, P = 0.39, I^2^ residual = 0.00%; for insulin: β = − 0.959, P = 0.19, I^2^ residual = 0.00%; for HOMA-IR: β = − 3.944, P = 0.12, I^2^ residual = 85.81) could resolve the observed heterogeneity for FBS and insulin.

### Meta-analysis of the effects of vitamin E supplementation or vitamin E along with omega-3 or magnesium supplementation on hormonal profile

**Figure 5:**
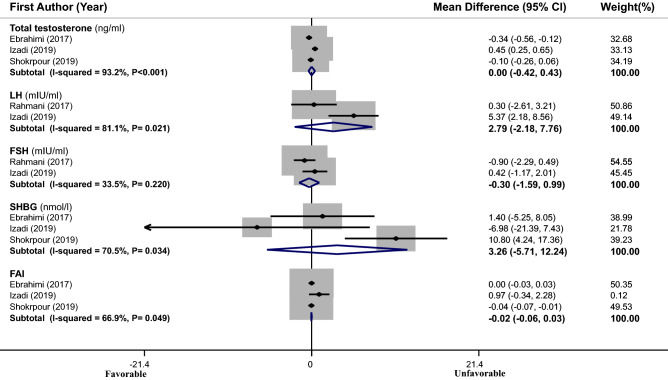
Forest plots of the effect of vitamin E supplementation or vitamin E along with omega-3 or magnesium supplementation on hormonal profile. If the diamond does not touch the vertical line (or the line of null effect), the overall effect is statistically significant. No significant effect of vitamin E supplementation or vitamin E plus omega-3 or magnesium supplementation on hormonal profile was found in PCOS patients.

Effect sizes of 3 eligible RCTs^14–16^ (with 171 subjects) were pooled; no significant effect for vitamin E supplementation or vitamin E along with omega-3 or magnesium supplementation was found [total testosterone (WMD = 0.00 mg/mL, 95% CI − 0.42 to 0.43), LH (WMD = 2.79 mIU/mL, 95% CI − 2.18 to 7.76), FSH (WMD = − 0.30 mIU/mL, 95% CI − 1.59 to 0.99), SHBG (WMD = 3.26 nmol/L, 95% CI − 5.71 to 12.24) and FAI (WMD = − 0.02, 95% CI − 0.06 to 0.03)] (Fig. 5). Between-study heterogeneity was significant in case of total testosterone (I^2^ = 93.2%, P < 0.001), LH (I^2^ = 81.1%, P = 0.021), SHBG (I^2^ = 70.5%, P = 0.034) and FAI (I^2^ = 66.9%, P = 0.049). Meta-regression based on age (for total testosterone: β = 2.195, P = 0.48, I^2^ residual = 99.36%; for SHBG: β = 0.094, P = 0.85, I^2^ residual = 0.00%; for FAI: β = 0.986, P = 0.71, I^2^ residual = 99.98%) and duration of intervention (for total testosterone: β = − 5.448, P = 0.19, I^2^ residual = 99.08; for FAI: β = − 3.923, P = 0.11, I^2^ residual = 95.63%) could not resolve the observed heterogeneity for total testosterone and FAI, while this analysis removed the observed heterogeneity for SHBG (β = 0.129, P = 0.79, I^2^ residual = 0.00%).

### Meta-analysis of the effects of vitamin E supplementation or vitamin E along with omega-3 or magnesium supplementation on anthropometric indices and hirsutism

As illustrated in Supplemental Fig. S1, vitamin E supplementation or vitamin E along with omega-3 or magnesium supplementation had no effect on weight (WMD = − 0.12 kg, 95% CI − 0.28 to 0.05), BMI (WMD = − 0.01 kg/m^2^, 95% CI − 0.07 to 0.05) and waist circumference (WMD = − 1.00 cm, 95% CI − 2.69 to 0.68) in PCOS patients. However, a significant decrease in hirsutism score (WMD = − 0.33, 95% CI − 0.65 to − 0.02) was observed among PCOS patients, after vitamin E supplementation or vitamin E along with omega-3 or magnesium supplementation as compared to placebo (Supplemental Fig. S2). There was no significant heterogeneity between studies in the case of hirsutism score and anthropometric measurements except for waist circumference (I^2^ = 91.3%, P = 0.001). Due to the small number of RCTs, subgroup analysis or meta-regression could not be performed for waist circumference.

### Sensitivity analysis and publication bias

Sensitivity analysis indicated that overall effect sizes for body weight and BMI variables did not substantially change after the elimination of each included study. Sensitivity analysis was not performed for other outcomes, due to the small number of eligible studies. Publication bias was not assessed, since the limited number of effect sizes (< 10 per each outcome) rendered the interpretation of the statistical tests unreliable^25^.

## Discussion

The current meta-analysis on 10 RCTs with 504 women with PCOS showed that vitamin E supplementation or vitamin E in combination with omega-3 or magnesium could result in significant decreases in lipid profile (TG, TC, LDL-c, VLDL, TC/HDL-c), hs-CRP and hirsutism score and an increase in NO concentration. Vitamin E supplementation or vitamin E, along with omega-3 or magnesium co-supplementation, had no impact on glycemic indices, hormonal profile, other biomarkers of inflammation or oxidative stress, anthropometric measurements, and HDL-c levels. So, the positive effect of vitamin E in PCOS might be independent of weight loss. Our findings highlighted the potential anti-hyperlipidemic, anti-oxidant, and anti-inflammatory properties of vitamin E on PCOS; therefore, this vitamin could be used as an adjunctive therapy for the management of some clinical manifestations and complications of PCOS.

Similar to our findings, a recent meta-analysis on RCTs showed that omega-3 and vitamin E co-supplementation could have beneficial effects on lipid profiles among overweight patients with metabolic syndrome^26^. In another meta-analysis on nine RCTs, omega-3 fatty acid supplementation among PCOS patients could decrease circulating TC, and TG concentrations^10^. Contrary to our findings, a meta-analysis on RCTs showed that vitamin E plus omega-3 fatty acid co-supplementation could reduce VLDL levels, but did not change other lipid indices among subjects with a wide range of metabolic disorders^27^. Also, in a meta-analysis of fifteen RCTs, it was shown that supplementation with tocotrienols did not reduce the concentrations of LDL-c, TC and TG of participants with various clinical conditions, while an increase in HDL-c levels was observed^28^. It is also worth noting that in most previous investigations, the term vitamin E was often synonymously used with α-tocopherol, while α-tocopherol is only one of eight natural forms of vitamin E, and new findings have suggested various properties for different forms of vitamin E^29^. In addition, among the investigations included in the present meta-analysis, only one study has specified the form of vitamin E as alpha-tocopheryl acetate^2^. In contrast, other articles did not specify the type of vitamin E supplemented with.

In line with our findings, a recent meta-analysis investigated the effects of vitamin E and omega-3 fatty acids co-supplementation on oxidative stress and inflammation among patients with different diseases and reported a significant decrease in hs-CRP and an increase in NO. Also, no significant effect on MDA or GSH was detected^30^. Similar to our findings, another meta-analysis on eight RCTs showed that vitamin E supplementation had no effect on BMI or insulin resistance (HOMA-IR) among patients with NAFLD; this investigation on NAFLD patients has also shown that vitamin E did not significantly change TG and TC levels, but decreased LDL-c concentrations^31^. Another meta-analysis on fourteen RCTs has documented no effect for vitamin E supplementation on glycemic profile (HbA1c, FBS, and fasting insulin) in patients with T2DM^32^. Although among all these mentioned investigations, the outcomes of interest (including metabolic syndrome, NAFLD, T2DM, and PCOS) were related to insulin resistance, these prior meta-analyses had different study populations, inclusion and exclusion criteria for eligible studies, type and dose of supplementation (vitamin E or omega 3 or vitamin E plus omega 3 co-supplementation), and gender of the study population. So, different methodology aspects led to different findings. More trials are needed to shed light on the effect of vitamin E supplementation on PCOS and other metabolic disorders.

NO is a signaling molecule with a large number of functions in the immune system, the nervous system and apoptosis. NO has additionally a key role in the pathogenesis of inflammation. It has an anti-inflammatory effect under normal physiological situations; whereas, it is considered as a pro-inflammatory mediator that induces inflammation due to overproduction in abnormal conditions^33^. This overproduction of NO as an inflammatory mediator can lead to tissue destruction such as in inflammatory autoimmune diseases. Therefore, NO is a ‘double-edged sword’ mediator; depending on the concentration, it has pro- or anti-inflammatory effects^33,34^. On the other hand, various established non-steroidal anti-inflammatory medications with NO releasing properties have been under intense clinical evaluations in the treatment of inflammatory disorders^35^. In case of women with PCOS, a previous meta-analysis on 12 case–control studies has documented that serum or plasma nitrite levels in patients with this syndrome were lower than healthy controls^36^. The current meta-analysis added the point that vitamin E supplementation alone and in combination with omega-3 or magnesium could significantly increase plasma NO levels in PCOS patients compared to controls. Therefore, this increment in NO concentration can be considered as an anti-inflammatory effect for NO in PCOS patients, although further investigations are required to understand the whole picture of such mediator.

Several mechanisms were suggested for the possible effects of vitamin E supplementation on various metabolic parameters. Chronic exposure to oxidative stress can impair lipid metabolism. Therefore, one hypothesis is that vitamin E improves lipid metabolism by reducing oxidative stress^18^. Anti-inflammatory and antioxidative effects of vitamin E may be explained by suppressing nuclear factor-kappa B (NF-κB) and JAK-signal transducer and activator of transcription 6 (STAT6) or JAK-STAT3 signaling pathways^37^. Moreover, tocotrienols can also downregulate the peroxisome proliferator-activated receptor gamma (PPARγ)^38^, which is a crucial mediator of adipogenesis^39^. Tocotrienols also possess dazzling lipid-lowering effects, such as downregulating 3-hydroxy 3-methylglutaryl coenzyme A (HMG-CoA) reductase and hence suppressing cholesterol synthesis^38^; hypocholesterolemic properties of tocotrienols are extended to inhibition of cholesterol absorption in the intestines, as well^40^.

There are several possible mechanisms underlying the association between vitamin E and glucose metabolism. Vitamin E, a fat-soluble antioxidant, can suppress reactive oxygen species (ROS) generation in the pancreas and maintain the structural integrity of pancreatic islets in experimental diabetes^41^. Furthermore, there are some evidence that vitamin E supplementation could inhibit the glycation of hemoglobin^32,41^ and partially reverse the beta-cell apoptosis caused by oxidative stress^41,42^.

The present systematic review and meta-analysis was the first one, to our knowledge, that assessed the effect of vitamin E supplementation or vitamin E in combination with omega-3 or magnesium on women with PCOS. We performed a methodologically strict systematic review of the literature. No evidence of substantial heterogeneity in this meta-analysis was found. However, our study has several limitations. The number of included RCTs was small, so we could not perform stratified analysis based on vitamin E vs. vitamin E plus omega 3 or magnesium supplementation. The included RCTs applied small sample sizes with relatively short intervention periods. Also, most of the eligible articles did not specify the type of vitamin E supplemented with. Finally, dietary intakes of vitamin E, omega-3 and magnesium were not considered in most included trials.

In conclusion, this study demonstrated that vitamin E supplementation or vitamin E in combination with omega-3 or magnesium co-supplementation might have beneficial effects on serum concentrations of TG, TC, LDL-c, VLDL, TC/HDL-c, hs-CRP, NO, and hirsutism score in PCOS patients. No significant effect was found in the case of glycemic indices, hormonal profile, anthropometric measurements, HDL-c levels, and other biomarkers of inflammation or oxidative stress. Additional well-designed clinical trials are needed to affirm these findings.

## Supplementary Information

Supplementary Information.

## Data Availability

The datasets used and analyzed during the current study are available from the corresponding author (Dr. Parvane Saneei at email: saneeip@yahoo.com) upon reasonable request.

## Abbreviations

PCOS: Polycystic ovary syndrome
T2DM: Type 2 diabetes mellitus
CVD: Cardiovascular diseases
WMD: Weighted mean differences
95% CI: 95% Confidence intervals
RCT: Randomized clinical trial
PRISMA: Preferred reporting items for systematic reviews and meta-analyses guideline
FBS: Fasting blood sugar
HDL-c: High-density lipoprotein-cholesterol
LDL-c: Low-density lipoprotein-cholesterol
TG: Triglycerides
TC: Total cholesterol
VLDL: Very low-density lipoprotein-cholesterol
MDA: Malondialdehyde
TAC: Total antioxidant capacity
GSH: Glutathione
hs-CRP: High sensitive C-reactive protein
NO: Nitric oxide
CRP: C-reactive protein
IL-6: Interleukin-6
TNF-α: Tumor necrosis factor-α
LH: Serum luteinizing hormone
FSH: Follicle stimulating hormone;
HOMA-IR: Homeostasis model assessment-estimated insulin resistance
QUICKI: Quantitative insulin sensitivity check index
SHB: Sex hormone-binding globulin
FAI: Free androgen index
BMI: Body mass index
WC: Waist circumference
METs: Metabolic equivalents
mf-G: Modified Ferriman–Gallwey
mg: Milligram
kg: Kilogram
μmol: Micro-mol
dL: Deciliter
IU: International unit
