# Supplementary material for: A meta-analysis of effects of vitamin E supplementation alone and in combination with omega-3 or magnesium on polycystic ovary syndrome
Source: Sci Rep. 2022 Nov 19;12:19927. doi: 10.1038/s41598-022-24467-0 (PMC9675810; doi:10.1038/s41598-022-24467-0)
Supplement: Supplementary file 1 — Supplementary Information. [file 41598_2022_24467_MOESM1_ESM.docx]

**Online Supporting Materials**

| Supplemental Table 1. Details of more relevant studies that were excluded. | | | |
| --- | --- | --- | --- |
| Reason of exclusion from current systematic review and meta-analysis | **Title of paper** | **Author/ Year** |  |
| Evaluated the effect of vitamin E on ovulation and pregnancy in women with PCOS | Randomized controlled open-label study of the effect of vitamin E supplementation on fertility in clomiphene citrate-resistant polycystic ovary syndrome | Morsy. A (1)/  2020 | 1 |
| A case report study | Can Vitamin E be a Key Supplementation in Polycystic Ovary Syndrome | Jayakumari. S (2), /  2019 | 3 |
| Evaluated the effect of a mix of vitamin E with other micronutrients in women with PCOS | The impact of a standardized micronutrient supplementation on PCOS-typical parameters: a randomized controlled trial | Hager. M (3)/  2019 | 4 |
| Evaluated the effect of short-term vitamin E supplementation on ovulation induction in women with PCOS | Effect of a short-term vitamin E supplementation on oxidative stress in infertile PCOS women under ovulation induction: a retrospective cohort study | Chen. J (4)/  2020 | 5 |
| Evaluated the effect of vitamin E on expression ratio of IL-1, IL-6, IL-8 and TNF-α and TGF-β gene in blood mononuclear cells in women with PCOS | The effects of omega-3 and vitamin E co-supplementation on parameters of mental health and gene expression related to insulin and inflammation in subjects with polycystic ovary syndrome | Jamilian. M (5)/  2018 | 6 |

REFERENCES:

1. Morsy AA, Sabri NA, Mourad AM, Mojahed EM, Shawki MA. Randomized controlled open‐label study of the effect of vitamin E supplementation on fertility in clomiphene citrate‐resistant polycystic ovary syndrome. Journal of Obstetrics and Gynaecology Research. 2020;46(11):2375-82.

2. Jayakumari S. Can Vitamin E be a Key Supplementation in Polycystic Ovary Syndrome. Indian Journal of Pharmacy Practice. 2019;12(2).

3. Hager M, Nouri K, Imhof M, Egarter C, Ott J. The impact of a standardized micronutrient supplementation on PCOS-typical parameters: a randomized controlled trial. Archives of gynecology and obstetrics. 2019;300(2):455-60.

4. Chen J, Guo Q, Pei Y-h, Ren Q-l, Chi L, Hu R-k, et al. Effect of a short-term vitamin E supplementation on oxidative stress in infertile PCOS women under ovulation induction: a retrospective cohort study. BMC women's health. 2020;20:1-9.

5. Jamilian M, Shojaei A, Samimi M, Ebrahimi FA, Aghadavod E, Karamali M, et al. The effects of omega-3 and vitamin E co-supplementation on parameters of mental health and gene expression related to insulin and inflammation in subjects with polycystic ovary syndrome. Journal of affective disorders. 2018;229:41-7.

**Supplemental Table 2.** Quality assessment of included studies

| Author (year) | | Random sequence generation (selection bias) | Allocation concealment (selection bias) | Blinding of participants and personnel (performance bias) | Blinding of outcome assessment (detection bias) | Incomplete outcome data addressed (attrition bias) | Selective Outcome reporting (reporting bias) | Other source of bias | Overall risk of bias |
| --- | --- | --- | --- | --- | --- | --- | --- | --- | --- |
| Jamilian, 2017 | Low risk | | Low risk | Low risk | Low risk | Low risk | **High risk** | Low risk | **High risk** |
| Rahmani, 2017 | Low risk | | Low risk | Low risk | Low risk | Low risk | Low risk | Low risk | **Low risk** |
| Ebrahimi, 2017 | Low risk | | Low risk | Low risk | Low risk | Low risk | Low risk | Low risk | **Low risk** |
| Izadi, 2019 | Low risk | | Low risk | Low risk | Low risk | Low risk | Low risk | Low risk | **Low risk** |
| Jamilian, 2018 | Low risk | | Low risk | Low risk | Low risk | Low risk | Low risk | Low risk | **Low risk** |
| Izadi, 2019 | Low risk | | Low risk | Low risk | Low risk | Low risk | Low risk | Low risk | **Low risk** |
| Talari, 2018 | Low risk | | **Some concerns** | Low risk | Low risk | Low risk | Low risk | Low risk | **Some concerns** |
| Shokrpour, 2018 | Low risk | | Low risk | Low risk | Low risk | Low risk | Low risk | Low risk | **Low risk** |
| Sadeghi ,2019 | Low risk | | Low risk | Low risk | Low risk | **High risk** | Low risk | Low risk | **High risk** |
| Shirazi, 2020 | Low risk | | Low risk | Low risk | Low risk | Low risk | Low risk | Low risk | **Low risk** |

**Supplemental Figure 1.** Forest plots of the effects of vitamin E supplementation or vitamin E along with omega-3 or magnesium supplementation on anthropometric measurements.

If the diamond does not touch the vertical line (or the line of null effect), the overall effect is statistically significant. No significant effects of vitamin E supplementation or vitamin E supplementation plus omega-3 or magnesium on anthropometric indices were found in PCOS patients.

**
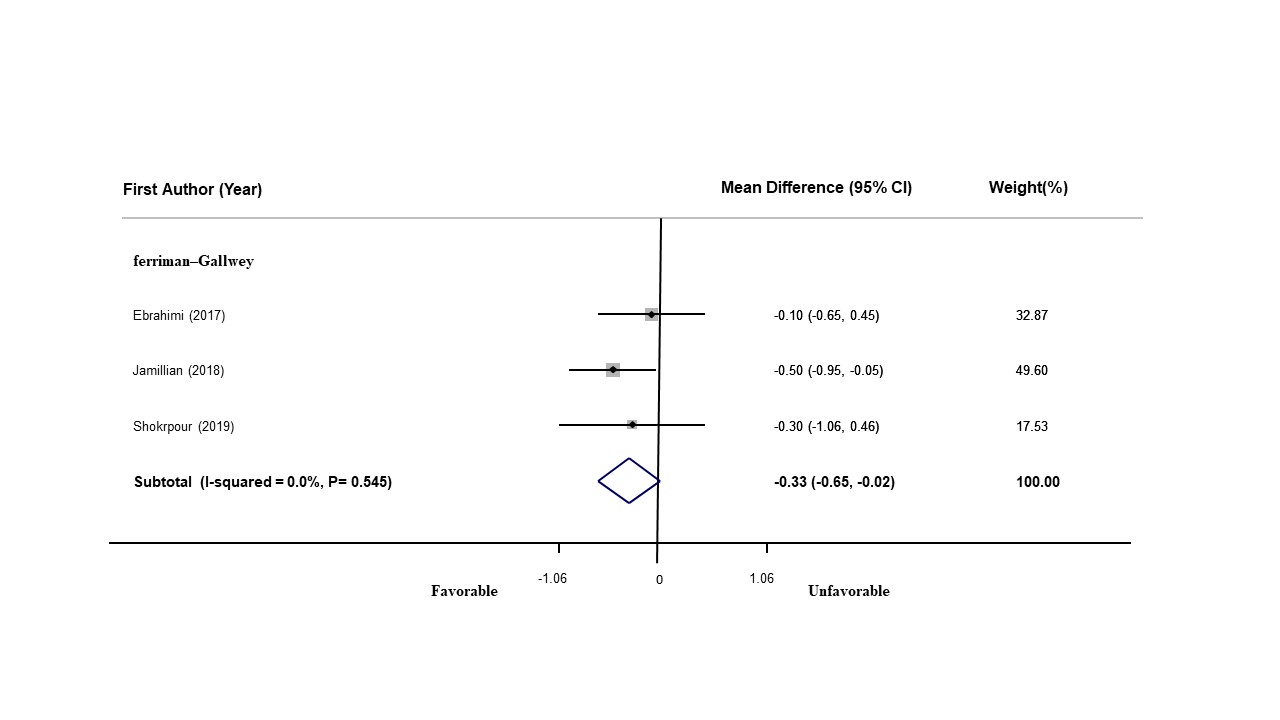
**

**Supplemental Figure 2.** Forest plots of the effects of vitamin E supplementation or vitamin E along with omega-3 or magnesium supplementation on hirsutism score.

If the diamond does not touch the vertical line (or the line of null effect), the overall effect is statistically significant. A significant decrease in hirsutism score (WMD= -0.33, 95% CI: -0.65 to -0.02) was found in PCOS patients after vitamin E supplementation or vitamin E along with omega-3 or magnesium supplementation in comparison to placebo.
